# Implementation process of community-based lifestyle interventions supported by technology for elderly population: qualitative evaluation of six European regions case studies

**DOI:** 10.3389/fdgth.2026.1905643

**Published:** 2026-08-14

**Authors:** Irati Erreguerena, Nerea González, Igor Larrañaga, Franco Mercalli, Eva Karaglani, Yannis Manios, Odysseas Androutsos, Julia Schellong, Kai Gand, Rosana Angles Barbastro, Maria Teresa Hurtado, Gloria Cea, Alba Gallego, Guiseppe Fico, Leandro Pecchia, Jordi de Batlle, Davide Piaggio, Ane Fullaondo

**Affiliations:** 1 Biosistemak Institute for Health Systems Research, Bilbao, Spain; 2 Network for Research on Chronicity, Primary Care, and Health Promotion (RICAPPS), Bilbao, Spain; 3 Research Unit, Barrualde-Galdakao Integrated Healthcare Organisation, Osakidetza-Basque Health Service, Usansolo, Spain; 4 Research Unit, Debagoiena Integrated Healthcare Organisation, Osakidetza-Basque Health Service, Arrasate-Mondragón, Spain; 5 MultiMed Engineers Srls, Parma, Italy; 6 Department of Nutrition and Dietetics, School of Health Science and Education, Harokopio University, Athens, Greece; 7 European Centre for Obesity, Harokopio University, Athens, Greece; 8 Lab of Clinical Nutrition and Dietetics, Department of Nutrition and Dietetics, School of Physical Education, Sport Science and Dietetics, University of Thessaly, Thessaly, Greece; 9 Department of Psychotherapy and Psychosomatic, Medical Faculty, Technical University Dresden, Dresden, Germany; 10 Unidad de Innovación, Servicio Aragonés de Salud, Barbastro, Spain; 11 Servicio Aragonés de Salud, Departamento de Sanidad, Zaragoza, Spain; 12 Life Supporting Technologies—UPM, Madrid, Spain; 13 Università Campus Bio-Medico, Roma, Italy; 14 Biomedical Research Institute of Lleida - Dr. Pifarré Foundation, IRBLleida, Centro de Investigación Biomédica en Red de Enfermedades Respiratorias (CIBERES), Lleida, Spain; 15 School of Engineering, University of Warwick, Coventry, United Kingdom

**Keywords:** community-based, ederly, healthy lifestyle, implementation, technology

## Abstract

**Introduction:**

Community-based interventions represent a strategic approach to integrating the salutogenic model, involving multi-sectoral stakeholders to improve the health of specific communities. In the context of the EU's ageing population, eHealth technologies provide valuable solutions by improving older individuals' health and well-being through better access to knowledge, strengthening environmental relationships, and supporting the sustainability of health systems. This manuscript explores a community-based health promotion intervention focused on eHealth apps tested across six European regions within the GATEKEEPER project.

**Method:**

An observational studies were conducted in the European regions of Basque Country (Spain), Aragon (Spain), Saxony (Germany), Puglia (Italy), Lodz (Poland), and Central Greece and Attica (Greece). Qualitative techniques were used to evaluate the implementation process of the community-based interventions, focused on the analysis of the facilitators, barriers, and contextual factors influencing delivery and end-users engagement to the technologies tested.

**Results:**

Several factors influenced the success of the interventions, including the customisation and adaptation of applications to users' specific needs, the provision of incentives to promote engagement, and the support from health and community professionals. Customising apps to be user-friendly and culturally relevant ensures accessibility for diverse populations, while adaptation addresses varying levels of health literacy and digital skills. Continuous support from professionals fosters trust, reduces barriers to adoption, and promotes sustained engagement.

**Discussion:**

This study provides insights into factors influencing the implementation process and adherence to digital health interventions. Understanding adherence, intention to use, and dropout rates is essential for identifying the factors contributing to the limited impact of digitally-enabled real-world interventions. The findings stress the importance of co-designing interventions, ensuring user involvement from the beginning, which improves the alignment of technology with users' needs and increases engagement.

## Introduction

The population aged 65 and over has increased in recent years, being expected to comprise 22% of the world's population by 2030 (1). In this scenario, the target life expectancy is 3.3 years longer for women than for men (2) in similar socio-economic conditions; however, women endure a greater burden of illness and discomfort (3). There are many complex factors (4) that constitute and influence ageing and can be categorised in two: factors related to the physiological risk of developing a disease and losing physical function; and psychosocial alteration determinants such as isolation, loneliness, depression, or lack of income (5–7)^,^. While healthcare systems are facing the challenges brought by this new paradigm of demographic change, this situation can also be seized as an opportunity to change the way care is delivered, facilitating services or deploying new resources for encouraging and improving elderly health treatment behaviours.

In this sense, social determinants (8, 9) can have a direct influence on health-related habits among this population (10). An example can be how the activity levels drop significantly when people retire: the promotion of active and healthy assets and the early detection of risk factors for functional decline is essential (11). Therefore, developing health promotion strategies (12), strengthening community-based interventions (13), and mobilising community health assets (14) to reinforce and adapt elderly people behaviours to healthier ones may be key especially when they still have a high and relatively stable functional capacity (15). The salutogenic approach (16, 17), which integrates health into all policies, and boosts health promotion actions and programmes, can be an appropriate model to be implemented in communities by considering people's health, well-being, and quality of life as a human right (18).

Community-based interventions constitute a strategy for integrating the salutogenic approach, by deploying actions where multi-sectorial stakeholders are involved (19) to improve the health status of a community or a group (20) in a specific area or setting (21). Furthermore, the main objective of community action is the “construction of citizenship”, strengthening their engagement to a community, or bolstering the empowerment, understood as the responsibility process of individual and collective people (22). The action focused on the community seeks to mobilise existing health assets (14) and include some main elements (23): (1) enhance communities: building community capacity to act together on health and the social determinants of health; (2) volunteer and peer-to-peer roles focused on enhancing the capacities of organisations and individuals; (3) foster collaborations and partnerships for the involvement of key stakeholder, and (4) give access to community resources: connecting people to resources for practical help, group activities and volunteer opportunities to meet health needs and increase social participation.

Health technologies can be effective instruments to address the challenge of ageing (24, 25) and to boost the community interventions for promoting health through the use of community health assets and/or resources (26, 27). Similarly, they allow progress to be made towards promoting the health of older people and tackling well-being outcomes, hence prevent, treat (28) and ensure the sustainability of health systems (16). In this sense, eHealth Apps offer the possibility of personalizing, tailoring and adapting behaviour change interventions based on real time user and environmental data to improve health outcomes (29). Therefore, these Apps become a potential tool to support health promotion, disease prevention and management of different pathologies and revolutionising the way that health professionals and researchers monitor, manage and promote population health (30).

As such, interest in mobile health apps is also driven by the possibility of replicating resource-intensive, face-to-face interventions for health promotion and management. However, despite the availability of many mobile health apps (71,000 new health apps were launched in 2020 (31), the level of adoption by end users is generally low and not sustained over time (55% of apps get less than 5,000 downloads per year and 62% of developers report digital solutions with less than 1,000 monthly active users (32). Additionally, in many eHealth studies, particularly those conducted on the Internet and especially with self-help applications, high dropout rates may be a natural and typical feature (33). Usage metrics and determinants of dropout should be highlighted, measured, analysed, and discussed. This also includes analysing and reporting on the characteristics of the population for whom the app ultimately “works”, i.e., those who remain in the trial and use it.

Despite the increasing development of community-based and digitally supported interventions aimed at promoting healthy ageing, existing evidence has largely focused on their effectiveness under specific conditions, with less attention paid to how these interventions are implemented across different real-world settings. Studies on mobile health and community-centred approaches highlight important variability in design, user engagement, and contextual adaptation, which can significantly influence outcomes (26, 29). In addition, high attrition rates and low sustained use of digital health solutions further underline the importance of better understanding implementation processes and user interaction in practice (34). However, there is still a lack of shared methodological frameworks to systematically evaluate the implementation, scalability, and transferability of community-based, technology-supported interventions across regions and populations (35). Addressing this gap is essential to identify key factors that influence successful deployment and long-term sustainability in diverse European contexts.

In this context the European Horizon 2020 project GATEKEEPER (Smart living homes—Whole interventions demonstrator for people at health and social risks) is a European multi-centric large-scale pilot on smart living environments (36) with the aim of tackling the improvement of the quality of life of citizens through advance Information and Communications Technologies (ICT). This manuscript describes a mixed-methods process evaluation of a community-based health promotion intervention deployed in six European regions within the GATEKEEPER project, focused on testing innovative eHealth Apps. The aim of this study was to evaluate the implementation of the intervention across settings and to identify the main facilitators, barriers, and contextual factors influencing its delivery.

## Methods

### Design

A longitudinal observational qualitative study was carried out in six different regions (Basque Country and Aragon from Spain, Saxony from Germany, Puglia from Italy, Lodz from Poland, and Central Greece and Attica from Greece) between 2021 and beginning of the 2024, in the framework or GATEKEEPER project (37), to evaluate the implementation process of the community-based lifestyle intervention, and the user experience regarding the technologies offered. Data analysis adheres to the Consolidated Criteria for Qualitative Research (Supplementary Material 1. COREQ checklist).

### Community-based lifestyle interventions

In the six implementation sites, there were implemented a community-based intervention based on self-managed digital health applications. The deployed interventions, the addressed target populations, and the digital solutions tested in the six implementation sites are described in Supplementary Material 2 & Table 1. By combining personalized content, unobtrusive monitoring, and coaching or guidance, they aimed to enhance independence, improve quality of life, and encourage active, and healthy ageing through digital, preventive, and user-centered strategies.

**Table 1:** Main characteristics of health promotion use cases of GATEKEEPER project.

	Basque Country	Aragon	Saxony	Puglia	Central Greece and Attica	Lodz
Intervention aim	Comprehensive health promotion	Lifestyle-related early detection	Mental health management	e-coaching	Metabolic syndrome prevention	Prevention for Non-adherence to medication
Digital solution	MAHA	MAHA	Intelli@ge	Activage	Metabolic Syndrome Management Platform	My Health Everyday
Platform	Mobile	Mobile	Mobile or web	Mobile	Web or Web + Wearables	Mobile
Mode of use	Self-managed	Self-managed	Self-managed	Self-managed	Self-managed	Self-managed
Target population	• ≥ 65 years • Caregivers	• ≥ 50 years • Risk of suffering chronic disease	• ≥ 50 years	• ≥ 55 years • Risk of robustness decay	• ≥ 55 years • Caregivers • Living at home • Risk factors of metabolic syndrome	• ≥ 50 years • Living at home • Asymptomatic or low symptomatic condition
Recruitment	Multi-channel	Multi-channel	Multi-channel	Multi-channel	Multi-channel	Multi-channel

In each site, an ecosystem composed of health care and community-based organisations, partners from industry, civil society, academia, and government authorities was built. Users were considered as participants if they met the inclusion criteria that were citizen over 50 years old, healthy or at risk of chronic diseases or mental impairment, and having agreed to take part in the intervention after being invited by the recruiters. Gender was not defined as an inclusion criterion, as these community-based interventions were designed to be inclusive and to reflect the diversity of the general population. In all cases, recruitment followed a multi-channel approach by coordinating the use of several communication and delivery pathways to engage participants and provide interventions and coordinating campaigns, both online and offline, through the platforms, resources and assets available to and used by several local partners and stakeholders. All the interventions designed obtained ethical approvals from their respective ethics committees.

### Participants

Stakeholders with different profiles that participated in the six interventions were recruited. By convenience sampling there were selected more than 100 people including healthcare professionals (doctors, nurses, pharmacists and managers) from Primary Care settings, social workers, community pharmacists, patients, several associations (e.g., home retirement clubs), city halls administrative personal and councillors, or community workers in charge of healthy aging programs or health promotion assets. Anyone who was actively involved in the interventions was invited to participate in the different qualitative activities conducted from March 2023 to February 2024. An introductory explanation was done during the first monthly meetings by the research team explaining their interest in exploring the community-interventions implementation process in different EU contexts.

### Data collection

Different qualitative techniques were implemented to gather stakeholders' perception on the intervention, usability, accessibility and satisfaction, as well as the usefulness and acceptance of the tools. Determinants that influence the commitment with and the adherence to the intervention in the different settings were explored as well. The activities were carried out by a researcher's team with qualitative techniques experience composed by PhD and senior researcher, female, and a BD junior researcher, female. This team was in charge to take field notes from all the activities carried out.

The activities conducted were:

1. Collective sense-making workshops (38) to identify barriers, strengths, key insights for improvement and sustainability of the use cases and share user experiences and ideas of new actions and activities to encourage users' engagement.
2. Participatory approach co-creation workshops (39) among intervention implementers and project managers to analyse the implementation methodologies, process, share baseline and intermediate results from each use case, Knowledge and experience exchange, and discuss activities conducted for plans redefinition. Moderated by one or more members of the large-scale pilot coordination team from Gatekeeper EU project, they took place in a hybrid environment having some participant's in-presence and others remotely.
3. Collaborative diagnostic sessions (40) for each region pilot to share and report the status of the interventions through the implementation log and issue tracker tool such as PowerBI (41) platform designed for this purpose and Warwick Analytics which pilots' managers completed to report key issues, propose mitigation actions, identify responsible partners, and schedule contingency plans.
4. Monitoring sessions for reporting Operative Key Performance Indicators (KPI) (42) through an ad-hoc Business Intelligence tool (i.e., Power BI) and Warwick Analytics, focused on enabling the measurement of the implementation and performance of the pilot, identify areas for improvement and facilitate the decision-making process. KPIs, Supplementary Material 3, were reported on monthly basis by pilots for assessing each pilot progress and identify needs and challenges to be addressed, users recruited, stakeholder engagement level, user registered on the tools, baseline questionnaire completed and others.

A summary of these KPIs by pilot site is provided in Table 2. However, these indicators reflect implementation processes (e.g., recruitment, engagement, and monitoring) and are not directly comparable with the qualitative findings presented, which capture stakeholders' perceptions of usability, engagement, and contextual barriers.

**Table 2:** Health promotion use cases of GATEKEEPER project implementation process KPIs.

	Basque Country	Aragon	Saxony	Puglia	Central Greece and Attica	Lodz
Total users involved	715	315	593	707	1,040	1,136
Recruited users	715	315	593	707	1,040	1,136
Contacted users	13,966	64	10,955	3,226	1,040	22,353
Expression of interest	9,045	64	593	939	1,000	22,353
Finalised users	0	0	0	434	864	460

Total users involved: Users that used the tool or technology implemented in the intervention.

Recruited users: users that meet the selection criteria and have signed consent forms.

Contacted users: users that were contacted or informed thought different dissemination channels to participate in the intervention.

Expression of interest: Number of users willing to participate.

Finalised users: users that completed the intervention.

In total, three collective sense-making workshops, one co-creation workshops, four collaborative diagnostic sessions and 10 monitoring meetings were conducted with the six pilot sites between March 2023 and February 2024. For each activity, structured documentation was produced, including meeting minutes, field notes, implementation reports and summaries of issues and decisions recorded in the Power BI and Warwick Analytics dashboards, which constituted the qualitative material used for thematic analysis.

### Data analysis

First, a descriptive analysis of each implementation site interventions was performed. The approach considered for the community-based interventions was based on an ‘intention to treat' approach, thus allowing the results to be analysed considering all people included in the study, regardless of whether they complied with the intervention protocol or not. The KPIs collected by each pilot site provided an unbiased estimate of the effect of the intervention, as it included compliers, non-compliers and dropouts.

The working data for the qualitative analyses consisted of: structured summaries and minutes from the collective sense-making and co-creation workshops generated by the project coordination team, monthly implementation logs and issue tracker outputs derived from the collaborative diagnostic sessions completed by each pilot in Trello and Etherpad platforms used for this activity, and monitoring reports and dashboards describing key performance indicators and contextual notes provided monthly by each pilot in an excel sheet template and published in PowerBI platform (42). These materials were treated as a single corpus and analysed jointly, allowing the integration of experiential accounts from stakeholders, implementation decisions and performance data narratives into a coherent thematic structure. All this information gathered in the documents developed was reviewed to describe the overarching scenario and become familiar with the data. Participants were not asked to provide feedback on the findings.

With these documents, an inductive and reflexive thematic analysis of narratives was carried out, by systematic reading, re-reading, and iterative re-coding. The thematic analysis is mainly described as “a method for identifying, analysing and reporting patterns (themes) within data” (41). The themes identified through the analysis structure the interpretation of findings in relation to the research question. Therefore, our aim was to find patterns of meaning and interpretive schemes, based on the themes that the participants use for making sentence of what was happening. The aim was also to transform the raw data into highly organised themes or categories. Initial codes were generated inductively from the data by the two members of the research team, who independently read and annotated the documents to identify recurrent patterns and salient issues related to implementation, user experience and contextual factors. Coding and data management were conducted manually, using structured coding matrices in spreadsheets. Codes were then grouped into candidate themes through team discussions, examining conceptual similarities and differences and ensuring that each theme captured a coherent pattern of meaning. Themes were iteratively reviewed against the coded data and the full dataset, refined for clarity and internal consistency, and agreed upon by the research team. The analytical process included data familiarization, initial coding, theme development, and iterative refinement of themes. Reflexivity was supported by regular analytic meetings in which researchers explicitly reflected on how their roles in the GATEKEEPER project, previous experience with digital health interventions and knowledge of each site might shape the interpretation of the data, and documented these reflections alongside analytic decisions. This process supported the confirmability by making explicit the influence of researcher perspectives on the analytical process.

Credibility was enhanced through the principal researcher's presence during the different activities and through data triangulation using the pilot site data sources, that is, all the documentation produced in the activities performed, to examine the consistency of the different data sources and results. Triangulation is a technique of comparing the information collected using different methods, to examine the consistency of different data sources and results (43). A comparison of the main results from different sites and sources supply additional observations, thus adding breadth and perspective and helping in understanding the outcomes. This approach contributed to the credibility and confirmability of the findings, while the multi-site design and contextual description of each pilot site support the transferability of the results. The analytical procedures were applied consistently across sites to strengthen dependability and to increase the transferability of the findings, the selection of diverse participants was ensured in terms of research experience, working background, and intervention involvement (44). All these procedures were followed to evaluate the trustworthiness of the study (45).

## Ethical considerations

The authors assert that all procedures contributing to this work comply with the ethical standards of the relevant national and institutional committees on human experimentation and with the Helsinki Declaration of 1975, as revised in 2008. All the interventions deployed in the six pilot sites obtained the Ethical Approval from their region/country and no human studies are presented in the manuscript. In the Basque Country region, it was approved by the Clinical Research Ethics Committee of the Basque Government (approval number PS2021007). In Greece, the Ethics Committees of Harokopio University (approval No.: C-2403/12-10-2020) and the Department of Nutrition and Dietetics of the University of Thessaly (approval No.: 2/30.11.2020) approved on of the studies, and it was registered at ClinicalTrials.gov (Identifier: NCT05031299). For Germany/Saxony: The study was approved by the Ethics Committees of Technical University Dresden BO-EK-30012021 “Gatekeeper Saxony mild—Prevention and early detection of mental health symptoms in the GATEKEEPER project—mental health stress and violence prevention in the 2nd half of life (taking into consideration COVID-19 effects)”. For Aragon region, Spain, the study was approved by the Regional Ethics Committee CEICA (Approval n° CI. PI20/383). For the Puglia intervention (Italy), the study was approved by the ethical committee of the Fondazione Casa Sollievo della Sofferenza Medical Science Department (Approval n° 857223). In Lodz region, Poland, the study was approved by the Komiskji Bioetycznej o Projekcie Eksperymentu Medycznego with the n° RNN/294/20/KE.

## Results

### Qualitative assessment

Qualitative data indicated that several factors influenced the implementation and uptake of digitally enabled community-based interventions across pilot sites. The qualitative findings are presented as pooled themes across sites, as the analysis aimed to identify common implementation patterns rather than to perform site-by-site comparisons, and because the available KPI data primarily reflect process indicators rather than outcome or effectiveness measures. These factors were grouped into five main domains: usability and technical performance, customization and engagement features, professional support, participant characteristics, and contextual barriers.

Usability and technical performance of the applications Low use of digital solutions was frequently associated with poor perceived usability, including difficulties navigating the interface, reaching content, and understanding how to use the applications. Participants also reported cumbersome download and registration processes, which in several cases required extensive personal information and the use of an email account that some users did not have. In addition, some applications were perceived as demanding too much effort each time they were accessed. These issues were consistently described as reducing initial uptake and regular use. For example, in the Basque Country pilot, workshop participants described the registration process as “too long and confusing”, noting that “many older users gave up when asked to create an email account”. Implementation reports similarly documented repeated support requests related to password recovery and navigation to basic functions, illustrating how technical complexity undermined sustained use among older adults.

#### Customisation, adaptation, and incentives of the applications

Several application characteristics that impacted on the recruitment numbers and user engagement. Stakeholders in co-creation workshops reported that “users quickly lose interest if the content does not change or respond to their progress”, and several pilot reports highlighted that the absence of tailored reminders and visible rewards reduced motivation to continue using the applications beyond the initial weeks. Some of the aspects that were highlighted in this sense were the little passive data collection such as sensors that capture food data or accelerometer data to capture physical activity, lack of individualised reminders, little adaptation of the contents offered to the user based on the interest, and no effective monetary rewards or incentives such as vouchers, lottery tickets or direct cash contributions for using the application.

By contracts, the integration of data collected through applications with existing health system technologies was found to enable health professionals to provide personalized recommendations that support improvements in users' health and daily lives. In addition, professional review of user activity and the content accessed contributed to the promotion of continued engagement with these technologies, which was reported as a positive feature in some pilot sites. This review also facilitated the personalization of information offered, allowing alignment with individual user interests and needs.

#### Personal support from health and community professionals

The presence of health or social care professionals during recruitment and follow-up was an important facilitator of engagement. Some specific aspects that intermediate in the community-based interventions as an important driver were the social characters, competitions and leader boards, community networking, network effects/peer pressure or the little communication between users and health professionals and/or citizen and community professionals. The unavailability of professional support and follow-up was associated with lower engagement and impacted in a negative way in the end-users interest in using the digital tools.

In contrast, participants who received regular message-based eCoaching from health or social care professionals frequently described the intervention as “feeling like someone cares about my health”, and monitoring notes from several pilots linked this perceived support to higher rates of continued log-ins and task completion.

#### Users' profile

The characteristics of the recruited population influenced both participation and retention. Low health literacy and low digital literacy were frequently linked to dropout or reduced engagement. The high age of the target population and the use of recruitment channels with limited reach among older adults, such as social media or online platforms, also negatively affected recruitment. These findings suggest that the recruitment strategy and the digital literacy level of the intended users are closely related to implementation outcomes. For instance, workshop discussions revealed that in some cases older adults unfamiliar with smartphones often relied on family members to install the applications, and some abandoned the intervention once this initial support was no longer available. Site reports also noted that recruitment campaigns conducted through social media reached relatively few of the oldest age groups, limiting participation among those most likely to benefit.

#### Other factors

Implementation was also influenced by contextual and organizational constraints. The high workload among primary care professionals, work stoppages related to holiday periods or strikes, and interruptions of face-to-face group activities due to the pandemic situation suffered because of COVID-19 hindered the intervention delivery in several sites. In some cases, the support offered to build partnerships for the interventions was considered inadequate or missing.

In addition, challenges were observed during user registration due to General Data Protection Regulation (GDPR) related registration procedures, which created challenges during user enrolment, and in some cases reduced trust and hindered the adoption of the applications. Participants' concerns about personal data use for research were also reported. In several sites, stakeholders reported that potential participants “did not want their data to be used for research” or felt uneasy about signing detailed GDPR forms, and pilot managers documented instances in which lengthy consent procedures led to drop-outs during enrolment. These examples illustrate how organizational constraints and data protection requirements directly shaped implementation dynamics.

## Discussion

This study provides real-life insights into the factors that influence adherence to a digital health intervention implementation process, including interventions implemented in community settings. By applying the same analytical procedures across heterogeneous pilot sites and documenting contextual particularities, the study was designed to explore which implementation lessons may be transferable across settings despite differences in specific technologies and target populations approached by interventions. The findings align with established implementation science frameworks, highlighting usability, professional support, and contextual adaptation as critical domains for technology-supported community interventions. Usability barriers such as complex registration and non-intuitive interfaces were consistently reported across sites, confirming that technical simplicity is a prerequisite for adoption among older adults with low digital literacy. Similarly, the positive role of professional support underscores the importance of hybrid models that combine digital tools with human interaction, particularly for populations at risk of isolation or functional decline. These insights provide practical guidance for designing scalable interventions that balance technological innovation with real-world feasibility. Despite the substantial heterogeneity in interventions, target populations and digital solutions across the six pilot sites, several implementation lessons appear broadly transferable. Core factors such as usability, availability of professional support, and alignment with users' digital literacy levels were reported as critical in all settings, suggesting that they should be considered foundational design principles regardless of the specific technology employed. Context-dependent elements, such as local governance structures, primary care workload or existing community assets, influenced how these principles could be operationalized in practice, underscoring the need for flexible adaptation rather than a one-size-fits-all model.

The learning from the pilot sites reinforces the need a co-design process (46) for intervention design as is being implemented in several contexts (47). User involvement in these interventions should rely on participatory co-creation, engaging end-users from the outset to ensure their needs and interests are addressed. Co-design fosters creativity, collaboration, and innovation within organizations while embedding users' capabilities and perspectives into the technologies developed (48). As a result, the digital solutions produced are more intuitive, accessible, and appealing, which increases user willingness to adopt and participate in the interventions.

The future of community-based health promotion interventions is increasingly digital, with mobile health apps emerging as powerful tools for supporting health promotion, disease prevention, and the management of various health conditions. These eHealth applications are revolutionizing how healthcare professionals and researchers monitor, manage, and promote population health, with the ultimate goal of enhancing overall well-being and care. However, as highlighted in the Gatekeeper project's community interventions, it is crucial that these applications are not only effective but also user-friendly, intuitive, and easy to navigate.

From a policy perspective, regulatory and governance frameworks are central to enabling the implementation, adoption, and scalability of digital health solutions. GDPR compliance emerged as a significant barrier to enrolment, particularly when procedures were perceived as complex or opaque. Participants expressed concerns about data use for research, which reduced trust and hindered uptake. These findings emphasize the need for simplified, transparent consent processes and proactive communication about data protection to build user confidence. In this context, the European Health Data Space represents a key policy development grounded in trust across Member States, which may facilitate the secondary use of health data by enabling standardisation, interoperability, and secure data sharing while maintaining strong privacy safeguards, thereby potentially supporting the broader scalability of digital health interventions across Europe (49).

Although the applications and innovative technologies provided integrated content and materials to improve users' quality of life and health, interaction, particularly among older adults, was limited. This low engagement led to poor adherence and high dropout rates, driven by factors that made sustained participation difficult.

As digital tools become central to community-based health promotion, it is vital to integrate health literacy into the design of eHealth applications. Health literacy influences how people access, understand, and use health information, making it a key factor in ensuring that technologies are both accessible and effective. Because users have varying levels of health literacy, involving them early in the design process helps tailor applications to their specific needs and capacities. This user-centred approach results in digital solutions that are intuitive, relevant, and more likely to be adopted, ultimately supporting better long-term health outcomes.

Evidence from the Gatekeeper pilots highlights the need for digital health tools that complement existing services, with participants favoring those offering individualized follow-up and personalized support. Recruitment can be strengthened by leveraging established solutions (50), integrating community initiatives (51), and conducting targeted campaigns (52), while incentives may further motivate engagement when users perceive clear benefits. Even effective technologies require adequate resources for dissemination, including marketing and awareness activities, to ensure visibility, trust, and sustained use. Overall, user-centered design combined with systematic recruitment and communication strategies is essential to maximize adoption and impact of digital health interventions

There are several articles (53, 54) in the literature that identify and typify some of the factors that influence adherence to health apps, focusing on the influence of the user's lifestyle. These aspects can be linked to intervention-related factors (e.g., features of the app itself, such as technical stability, customisation, registration or personal support) or user-related factors (e.g., user profile in terms of social determinants, health education or digital literacy). Therefore, understanding the factors that influence in the implementation process and engagement of the end-users with the intervention, including community-based interventions based on digital applications, is a key factor in understanding and preventing user attrition and can affect the effectiveness of digital health interventions (55).

Key learnings for implementation and scale-up include: (1) prioritize usability testing with end-users during development to minimize technical barriers; (2) integrate professional support and eCoaching as core components rather than optional features; (3) adapt recruitment strategies to the digital literacy and preferences of older adults; (4) allocate resources for partnership development and contextual adaptation across sites; and (5) simplify data protection processes to enhance trust. Taken together, these key learnings represent implementation strategies that are likely to be transferable across European community settings, as they address common barriers identified in heterogeneous contexts while allowing for local tailoring of specific tools and workflows.

Barriers outlined in this study can be opportunities for future technology-enabled community interventions, so technology uptake and scalability are maximized. Recognising the diversity of the population when developing and implementing digital technologies has the potential to increase the impact, engagement and adherence to the intervention. By explicitly accounting for population diversity and contextual particularities, the transferable lessons identified in this study can guide the adaptation of digital community-based interventions to new settings while maintaining their core implementation components.

### Limitations

Research on older adults' use of health technologies frequently relies on implicit inclusion criteria tied to technology use, potentially leading to significant selection bias. In the present study, participants were recruited through a multi-channel strategy that may have led to the inclusion of more motivated, health-conscious, digitally literate, and technology-oriented individuals., while underrepresenting older adults with lower digital literacy or limited access to technology. This limits the transferability of the findings to the broader older adult population and should be considered when interpreting the reported levels of usability, usefulness, and acceptability.

The socio-cultural diversity of the contexts in which health technologies were implemented also represents an important limitation. Regional differences in cultural values, health practices, lifestyles, digital literacy, healthcare infrastructure, and access to technology can influence older adults' engagement with interventions and their responses to benefits, potentially limiting the transferability of findings across settings.

Another important consideration relates to the nature of the data collection process. Much of the qualitative information was obtained through workshops, stakeholder discussions, and reports involving participants and professionals who were actively engaged in the interventions. Their close involvement may have introduced social desirability bias, potentially leading to more favorable assessments of the technologies and implementation processes. In addition, the study relied primarily on self-reported perceptions and experiences, without incorporating complementary observational methods, such as participant observation or direct behavioral assessments that could have helped triangulate findings and strengthen the robustness of the interpretations. Furthermore, the qualitative data were not collected directly from end users but from stakeholders involved in the implementation process, who reported perceptions and feedback gathered from participants. This indirect perspective may limit the ability to capture first-hand user experiences and should be considered when interpreting the findings.

Finally, the use of different health applications across pilot sites complicates the comparability and interpretation of results, as variations in design, content, and functionality influence how older adults interact with and benefit from the technology. Differences in usability, usefulness, and acceptability must be interpreted within each pilot's context, underscoring the need to tailor health technologies to older users and to contextualise findings across settings.

## Conclusions

The Gatekeeper community-based interventions highlight the need for health technologies that are effective, accessible, and user-friendly for older adults. Engaging this population from the earliest stages through participatory co-design ensures that tools reflect their abilities, preferences, and needs, thereby enhancing usability and sustained engagement. Successful enrolment is further supported when technologies are prescribed or endorsed by trusted healthcare and community professionals, whose guidance and ongoing assistance foster legitimacy and help overcome scepticism. Simplified interfaces and targeted education that address varying levels of health literacy empower users to manage their health more independently. Taken together, a holistic approach that combines user-centered design, health literacy support, and professional involvement is essential to maximize adoption, long-term engagement, and the overall impact of digital health interventions among elderly populations.

## Data Availability

The datasets presented in this article are not readily available because the health organization is the owner of the dataset. Requests to access the datasets should be directed to irati.erreguerenaredondo@bio-sistemak.eus.

## Group member of GATEKEEPER consortium

Mª Luz Peña, Begoña Belarra, Mª del Puy López, Idoia Sanz Murua and Esteban de Manuel Keenoy from Osakidetza Basque Health Service organization, Basque Country pilot site, Spain. Ivana Lombroni, Eugenio Gaeta, and The multidisciplinary team Life Supporting Technologies, Universidad Politécnica de Madrid (UPM), Spain. Organizations AReSS (Bari), Fondazione Politecnico di Milano (Milano), Casa Sollievo della Sofferenza (San Giovanni Rotondo), InnovaPuglia (Bari) and MultiMed Engineers (Parma) from Puglia pilot site, Italy. Yannis Manios, Eva Karaglani, Ilektra Kalogerakou, Alexandros Karagiannis, Ioanna Nakaki, Elpiniki Vlachopoulou, Maria Dimopoulou, Maria Skaltsa, Ioannis Chrysou, Konstantinos Leontiou, Karamperi Katerina, Evangelia Malakou, Anastasios Papalazarou, Elisavet Parlapani, Nasia Paschaleri, Christos Paximadas, Maria-Sofia Pelagidou, Kyriacos Reppas, Katerina Tyrothoulaki, Konstantinos Xenos, Spyridon Zarogiannis, Konstantinos Zervos, Rafaela Makri from Harokopio University (Greece). Odysseas Androutsos, George E. Dafoulas, Alexandra Bargiota, Thomas Tzekos, Athanasios Topis, Nikolaos Papaspanos, Ifigeneia Evangelia Chionidi, Mylona Eleni, Yvoni Katergari, Vaia Katsarou, Anastasia Davitidou, Glykeria Papagiannopoulou, Alexandra Koumpitski, Karaiskou Avgi, Iliana Evangelou from the University of Thessaly (Greece). Nico Schultz and Hannes Schlieter from the Department of Psychotherapy and Psychosomatic, Medical Faculty, Technical University Dresden, Germany.
