# Supplementary material for: Implementation process of community-based lifestyle interventions supported by technology for elderly population: qualitative evaluation of six European regions case studies
Source: Front Digit Health. 2026 Aug 14;8:1905643. doi: 10.3389/fdgth.2026.1905643 (PMC13522155; doi:10.3389/fdgth.2026.1905643)
Supplement: Supplementary file 1 [file Datasheet1.pdf]

Community-based lifestyle interventions short descriptions[1][i]:

Basque Country (Spain)

In Basque Country the intervention aimed to improve healthy habits and health quality by promoting the use of a health promotion technology. It was based on self-managed Movement for Active and Healthy Aging (MAHA) mobile application[2]. The target population was people over 65 years old and/or their caregivers. The aim was to 1) encourage active and healthy ageing, promoting physical activity, cognitive training and social participation 2) enhance independence, autonomy and improve the well-being and 3) increase the empowerment. For that purpose, educational materials and contents on health were made available in the application, as well as recommendations for activities to promote healthy lifestyle habits.

Aragon (Spain)

In Aragon the intervention was focused on a lifestyle-related early detection and intervention service. It was also based on self-managed MAHA mobile application. The target population was people over 50 years old, having a potential risk of suffering from a chronic disease and who had signed the informed consent. The aim was 1) to avoid/delay/prevent the appearance of chronic disease, 2) to empower patients on the self-management of their disease and 3) to promote healthy habits. For that purpose, the tool provided content and services best suited to each user's profile, sending tips and suggestions for a healthy lifestyle and analysing behavioural patterns to detect a sedentary lifestyle or isolation. A previous consent form signature was required for ensure the patient identification, to protect the user’s data and provide personalized advice, integrated with the electronic healthcare record.

Saxony (Germany)

In Saxony the intervention was centred on a mental health managing service. It was based on the stand-alone and self-managed Intelli@ge mental health application[3], which can be used as a mobile application or web application. The target population was people over 50 years old and who have signed the informed consent. The aim was to 1) detect and manage mental health symptoms, improve self-management for daily coping and facilitate mental healthcare use, 2) enhance the daily activity and social participation and 3) assess the COVID-19 related burden. For that purpose, the application offers information on general and mental health, self-assessments and exercises for relaxation, stress reduction and mindfulness, as well as contact addresses to get help.

Puglia (Italy)

In Puglia the intervention aimed to deploy an e-coaching service. It was based on the self-managed Activage mobile application. The target population was healthy people over 55 years old, at risk of decay. The aim was to 1) early detect signs of decay, 2) implement a community health promotion scheme and 3) maintain an active and healthy lifestyle. For that purpose, the tool enabled to unobtrusively monitor deviations in daily activity and habits, share the information with participants and people they trust and deliver one-way coaching messages to encourage participants to adopt healthy behaviours.

Lodz (Poland)

In Lodz the intervention was based on the prevention of non-adherence to medication prevention service. It was based on the self-managed My Health Everyday mobile application[4]. The target population was people over 50 years old, living at home and at least one asymptomatic or low symptomatic conditions between hypertension, congestive heart failure, hyperlipidaemia, diabetes, hypothyroidism, chronic obstructive pulmonary disease (COPD) and/or chronic kidney disease. The aim was to 1) lower the risk of medication non-adherence 2) lifestyle modifications, promotion of healthy habits and better coping with health management, 3) ensure healthy ageing and maintain quality of life, 4) increase health literacy and awareness and 5) safe detect and future protect from medication non-adherence. For that purpose, the tool enabled an unobtrusive behaviour monitoring for early detecting the risk of non-adherence and the implementation of e-coaching interventions.

Central Greece and Attica (Greece)

In Central Greece the intervention aimed to evaluate a metabolic syndrome prevention service. Two interventions were deployed, one based on self-managed Metabolic Syndrome Management Platform web application, and the other based in the web application plus Aria Air smart weight scale and Fitbit Inspire 2 activity tracker. The target population was people over 55 years old and/or their caregivers, living at home, at risk of metabolic syndrome and who signed the informed consent. The aim was to 1) measure the effect on waist circumference reduction and 2) assess changes in body mass index (BMI), percentage body fat, patient-reported outcome measures (PROMs), physical activity increase and sedentary time decrease, sleep quality and quality of life. For that purpose, the tool enabled healthcare professionals to create and assign personalised care plans and track the dietary habits, exercise time and medical records of each participant.

[1] de Batlle J, Benítez ID, Moncusí-Moix A, Androutsos O, Angles Barbastro R, Antonini A, Arana E, Cabrera-Umpierrez MF, Cea G, Dafoulas GE, Folkvord F, Fullaondo A, Giuliani F, Huang HL, Innominato PF, Kardas P, Lou VWQ, Manios Y, Matsangidou M, Mercalli F, Mokhtari M, Pagliara S, Schellong J, Stieler L, Votis K, Currás P, Arredondo MT, Posada J, Guillén S, Pecchia L, Barbé F, Torres G, Fico G; GATEKEEPER project. GATEKEEPER's Strategy for the Multinational Large-Scale Piloting of an eHealth Platform: Tutorial on How to Identify Relevant Settings and Use Cases. J Med Internet Res. 2023 Jun 28;25:e42187. doi: 10.2196/42187. PMID: 37379060; PMCID: PMC10365628.

[2]https://maha.lst.tfo.upm.es/#:~:text=%C2%BFQu%C3%A9%20es%20MAHA?,desplegamos%20en%20sus%20propios%20entornos.

[3] https://www.gatekeeper-project.eu/blog/mental-health-50plus-the-gatekeeper-saxony-pilot/

[4]https://www.gatekeeper-project.eu/blog/helping-patients-adhere-to-their-medical-treatment-with-a-mobile-app-that-only-needs-one-minute-of-your-time-2/
